# Broad-Spectrum Activity of Small Molecules Acting against Influenza a Virus: Biological and Computational Studies

**DOI:** 10.3390/ph15030301

**Published:** 2022-02-28

**Authors:** Mariangela Agamennone, Fabiana Superti

**Affiliations:** 1 Department of Pharmacy, University “G. d’Annunzio” of Chieti-Pescara, Via dei Vestini 31, 66100 Chieti, Italy; m.agamennone@unich.it; 2 National Centre for Innovative Technologies in Public Health, National Institute of Health, Viale Regina Elena 299, 00161 Rome, Italy

**Keywords:** Influenza, hemagglutinin, neutralization, homology modeling, docking, small molecules, receptor binding site, molecular dynamics

## Abstract

Influenza still represents a problematic disease, involving millions of people every year and causing hundreds of thousands of deaths. Only a few drugs are clinically available. The search for an effective weapon is still ongoing. In this scenario, we recently identified new drug-like compounds with antiviral activity toward two A/H1N1 Influenza virus strains, which were demonstrated to interfere with the processes mediated by hemagglutinin (HA). In the present work, the compound’s ability to act against the A/H3N2 viral strain has been evaluated in hemagglutination inhibition (HI) assays. Two of the five tested compounds were also active toward the A/H3N2 Influenza virus. To validate the scaffold activity, analogue compounds of two broad-spectrum molecules were selected and purchased for HI testing on both A/H1N1 and A/H3N2 Influenza viruses. Forty-three compounds were tested, and four proved to be active toward all three viral strains. A computational study has been carried out to depict the HA binding process of the most interesting compounds.

## 1. Introduction

Flu is a contagious respiratory disease caused by Influenza viruses that infect the nose, throat, and sometimes the lungs, causing seasonal epidemics with potentially severe outcomes. Viral infection can cause mild to severe illness and can lead to death at times. Influenza virus infections are a significant cause of morbidity and mortality worldwide, mainly among aged and high-risk people [1]. Before the dramatic emergence of the SARS-CoV-2 virus, pandemic Influenza has always been a growing threat to public health worldwide. In the 20th century, three outbreaks of human pandemic influenza occurred in 1918, 1957, and 1968, causing significant mortality. Notably, an A/H1N1 strain caused the 1918 pandemic, Spanish Flu, infecting about 500 million people and killing 50 million [2]. Over the past two decades, Influenza A virus H1N1 2009 (A/H1N1/pdm09) caused the first Influenza pandemic of the 21st century. The new H1N1 virus was detected first in Mexico and the United States [3] and spread quickly across the world, affecting over 214 countries and causing over 18,449 deaths [4]. On 10 August 2010, the World Health Organization (WHO) declared the end of the 2009 global H1N1 influenza pandemic. However, the virus circulates as a seasonal Flu virus and causes illness, hospitalization, and deaths worldwide every year. A study on the impact of Influenza on excess mortality in all ages in Italy in recent seasons (season 2013/14–2016/17) reported that over 68,000 deaths were attributable to Influenza epidemics [5]. According to data collected by the Centers for Disease Control and Prevention (CDC) from 2010 to 2020, Flu was estimated to have caused 12,000–52,000 deaths annually. During that time, Flu also caused 9 to 41 million illnesses and 140,000–710,000 hospitalizations. Globally, the WHO has estimated that seasonal Influenza may result in 290,000–650,000 deaths each year due to respiratory diseases alone [6]. Several studies on factors linked with high influenza-associated respiratory mortality showed that mortality was higher in the seasons dominated by subtype A/H3N2 in people over the age of 65, but higher in seasons dominated by A/H1N1 in people aged 65 or younger [7,8,9,10]. The repeated episodes of Flu result from antigenic drift or antigenic shift that cause strain and subtype changes of Influenza viruses, respectively [11,12].

The preferred strategies to fight Influenza virus infection are vaccination and antiviral drug treatment. Vaccination is the primary means to prevent and control seasonal Flu; however, the success of the vaccine may vary based on different factors, including the time needed to design and produce the vaccine, limited worldwide availability, and, in particular, the genetic relationship between the viruses utilized for the vaccine and the circulating strains. Universal intervention methods against different Influenza virus subtypes have been explored to address the problems of antigenic variation [13,14,15]. 

Antiviral drugs may represent another option as universal intervention methods to fight the different viral subtypes. As of today, only three classes of chemotherapeutic drugs have been approved by Food and Drug Administration (FDA) and are available for the influenza virus infection treatment: the matrix protein 2 (M2) inhibitors amantadine (generic) and rimantadine (Flumadine^®^ and generic), the neuraminidase (NA) inhibitors oseltamivir phosphate (available as a generic version or under the trade name Tamiflu^®^), zanamivir (trade name Relenza^®^), peramivir (trade name Rapivab^®^), and the cap-dependent endonuclease (CEN) inhibitor baloxavir marboxil (trade name Xofluza^®^). Baloxavir is not recommended for pregnant women, breastfeeding mothers, outpatients with complicated or progressive illness, or hospitalized patients as there is no information about its use in these patients. Unfortunately, over the last years, Influenza viruses are showing, in a threatening manner, resistance phenomena associated with the overwritten drugs. As an example, all currently circulating Influenza viruses are resistant to adamantane antiviral drugs, and these are therefore not recommended for monotherapy. To circumvent this issue, efforts are made to develop new strategies or therapeutic approaches by researchers. The declared goal to fight seasonal Influenza remains the vaccine, even if its development proceeds slowly due to several factors. Therefore, through the search for new antiviral drugs [16,17,18,19,20], the use of specific monoclonal antibodies [21,22] and the development of combination therapies [23,24,25] antiviral strategies with alternative mechanisms of action towards different targets are studied. Some of these antivirals are small molecule drugs that, in contrast to antibodies, offer the advantage of oral bioavailability, high shelf stability, standardization, and relatively low production costs. Some examples are represented by new neuraminidase, cap-binding, and polymerase inhibitors [26,27,28].

**Figure 1:**
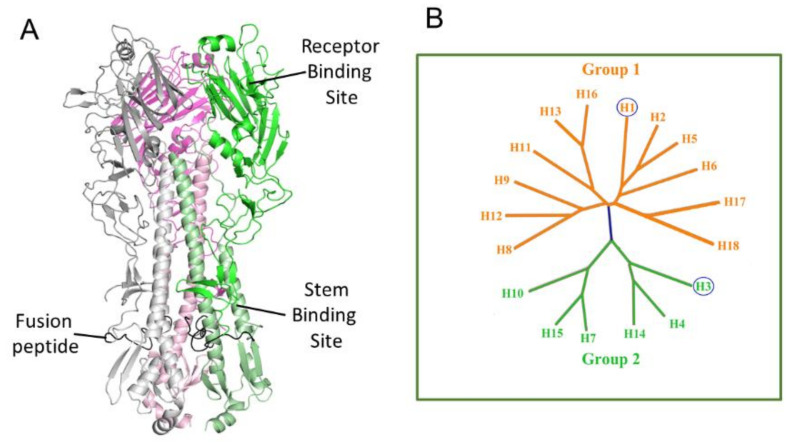
(**A**) Structure and (**B**) phylogenetic tree of hemagglutinin.

Among the protein targets of new anti-influenza drugs, many studies have considered viral hemagglutinin (HA) for its role in virus adhesion and internalization [29]. HA is a surface glycoprotein responsible for the binding of the virus to host cells and subsequent membrane fusion within the late endosome. HA is a 75–80 kDa homotrimer, and each monomer is composed of two polypeptides, HA1 and HA2, where the HA1 subunit forms mainly the head region of HA, while the HA2 subunit is the primary component of the stem region (Figure 1A). Eighteen types of HAs have been characterized and can be divided into two groups based on phylogenetic development and sequence similarity. Each group can be split into more clades (Figure 1B). 

The critical role played by this glycoprotein has prompted the development of antibodies directed toward the conserved stem region of HA to generate a universal vaccine [30,31,32]. Furthermore, many small molecules have been developed blocking the viral entry [33]. These latter can be important tools in Flu prevention and treatment as no FDA-approved therapy specifically blocks receptor binding or fusion mechanism. Umifenovir (Arbidol) is an orally active antiviral small molecule used to treat Influenza in Russia and China [34]; however, this HA targeting inhibitor is not approved for use in other countries, and large doses are needed to achieve therapeutic efficacy [35]. While Arbidol has broad-spectrum antiviral activity against different pathogenic viruses [34], it has recently demonstrated its binding to a specific hydrophobic cavity in the upper region of the HA stem [36]. Notwithstanding, Arbidol needs comprehensive optimization to improve HA affinity and pharmacokinetic stability, and additional molecules targeting HA on other surface epitopes need to be identified.

In our research efforts against Influenza, we focused on hemagglutinin as the target of lactoferrin and its derived peptides [37,38,39,40]. Furthermore, we recently published the identification of new drug-like compounds with anti-Influenza activity toward two A/H1N1 strains [41]. They showed a sub-micromolar activity in hemagglutination inhibition (HI) assays, neutralization and hemolysis studies. All activities indicate that they can block the adhesion to the host cell by hemagglutinin.

In the present work, the compound’s ability to act against A/H3N2 viral strain has been evaluated in HI assays to test if they can block the clinically relevant HAs. A/H1N1 and A/H3N2 strains are often present in seasonal Influenza circulating every year around the world. These HAs, moreover, are phylogenetically far from each other (Figure 1B), and therefore inhibition of both A/H1N1 and A/H3N2 accounts for a broad-spectrum activity. 

Two out of five previously tested compounds showed activity toward the A/H3N2 strain. To validate the scaffold antiviral property, analogue of the two hits were selected and purchased for HI testing on three viral strains. Forty-three compounds were tested, and four of them were active toward all three strains.

**Figure 2:**
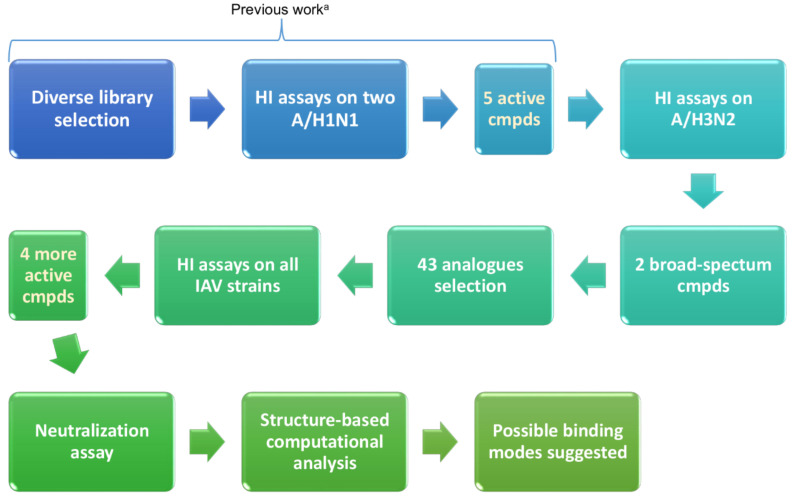
Schema reporting the step-by-step procedure applied. ^a^ This part of the workflow is reported in a previous article [41].

A comprehensive computational study has been carried out using both docking and molecular dynamics (MD) calculations to better depict the interaction between these molecules and target HAs, providing a suggestion regarding their mechanism of action. Structure-based approaches are often applied to study the binding of proteins, peptides, and small molecules interacting with HA [42,43,44,45,46]. The schema of the different phases of the work is summarized in Figure 2.

## 2. Results

### 2.1. Hemagglutination Inhibition (HI) Test against the A/H3N2 Strain

In the previous article, a set of five active small molecules has been identified. In the present study, these compounds have been assayed toward the A/H3N2 strain in order to evaluate their ability to block the interaction between the HA and the target cells in HI assays. Results obtained are shown in Table 1. Two out of the five tested compounds presented a sub-micromolar potency toward A/H3N2, demonstrating a large spectrum of activity.

### 2.2. Compound Scaffold Validation

A set of analogues for each hit was selected through substructure search in the Enamine database to validate the scaffolds of broad-spectrum compounds (scaffold structures used in the search are reported in the Supplementary Materials). Available compounds were tested in HI assays toward the three studied HAs.

The new set of assayed compounds comprised 22 analogues of reference hit **1,** and twenty-one of **4**, resulting in forty-three molecules. A total of 23 compounds out of 43 showed activity on at least one of the studied viral strains: 19 compounds were active against the oseltamivir-sensitive strain (A/Roma/H1N1), 19 were active against the oseltamivir-resistant strain (A/Parma/H1N1), and 16 on both H1N1 strains. These analogue compounds were all more active than the original hits toward A/H1N1 strains reaching a picomolar activity (Table 2).

Five compounds were effective against the A/H3N2 strain, and four proved to be active towards all three tested strains. In particular, one compound (**13**) presented an excellent ability to interfere with hemagglutinin binding to the host cell glycoproteins. Concerning the reference scaffold, nine active compounds are analogues of **1**, while 14 are derived from hit **4**. Moreover, both scaffolds (the imidazolyl-phenyl-sulfonamide and the pyridyl-carboxyl) are represented in the broad-spectrum active compounds. These data confirm the validity of both scaffolds in inhibiting hemagglutination of phylogenetically far hemagglutinins.

### 2.3. Neutralization Assays

Neutralization studies have been carried out on the newly identified four broad-spectrum compounds toward all Influenza A strains, while hits **1** and **4**, that were been already tested against A/H1N1 strains [41], were assayed in this work toward A/H3N2 to complete their activity profile. The neutralization test, in fact, detects the overall antiviral activity, since, by providing information on the titer needed to block the cytopathic effects of the virus, it allows identification of antiviral molecules able to block the entry of the virus into the cell, the viral internalization, and the fusion of the HA. Results obtained are shown in Table 3.

Compounds **1** and **4** showed the most interesting activity profile reaching the lowest EC_50_ toward A/Parma/H3N2 and A/Roma/H1N1, respectively, and the highest selectivity index. Ligand **4**, in particular, presents the best effectiveness toward the H1N1 strains. The other compounds that were more potent than primary hits in the HI assay, maintain a sub-micromolar potency and an acceptable selectivity index. Even though further optimization studies are necessary to obtain the best candidate, these results are of great value for developing new anti-Influenza virus compounds.

### 2.4. Computational Studies

As already verified for the previously identified compounds, biological assays suggest that these ligands interfere with HA functions.

**Figure 3:**
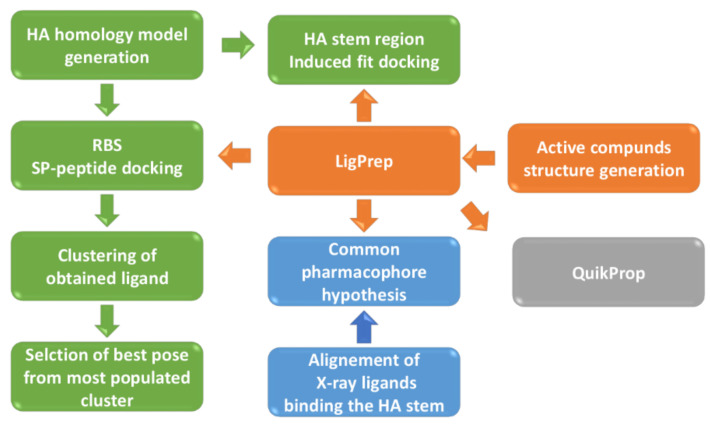
Schema reporting the step-by-step procedure applied in the computational studies.

To rationalize the experimental activity data, we carried out a structure-based computational study to depict the binding process between these ligands and hemagglutinin (Figure 3). It is well-known that HA is a large homotrimer, and each monomer is constituted by HA1 and HA2 chains. The HA1 sequence mainly forms the globular head of the mushroom-shaped protein where is located the receptor-binding site (RBS), while the HA2 chain forms the more conserved stem region that contains the fusion peptide, a sequence of 15 amino acids responsible for the pH-triggered conformation rearrangement of HA (Figure 1A).

In our study, the 3D structures of studied HAs were obtained by homology modeling, exploiting the SwissModel tool, and starting from the sequences of the HA strains used in the experimental assays, as already described in a recently published article [39].

The HI assay assesses the compound’s ability to interfere with the recognition of sialic acid of host cell glycoproteins by hemagglutinin [47]; therefore, the binding in this site was evaluated by docking calculations. This interaction occurs in the Receptor Binding Site (RBS), a more conserved region on the hypervariable Receptor Binding Domain (RBD), located on the globular head of HA [48]. 

All active ligands were docked in the RBS of each HA, exploiting a protocol which has already been set up, aimed to broadly explore the ligand conformations fitting the binding site [39]. The RBS is quite solvent-exposed, and several possible binding geometries can be retrieved. The applied protocol aims at expanding the conformational sampling to identify the most plausible binding modes.

**Figure 4:**
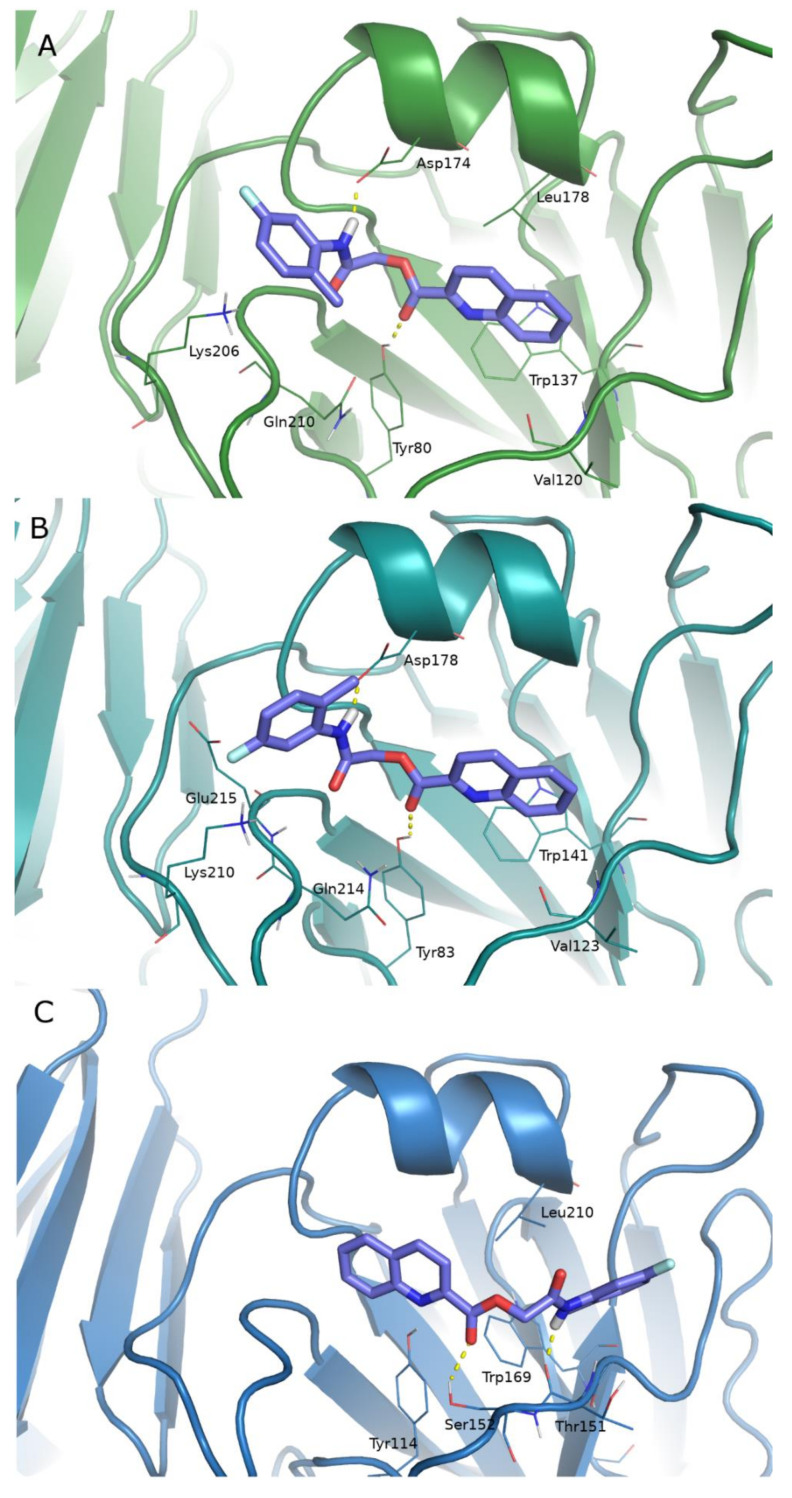
Docked pose of ligand **4** (stick, violet C atoms) in the RBS of the HA of A/Parma/H1N1, green cartoon (**A**), A/Roma/H1N1, cyan cartoon (**B**), and A/Parma/H3N2, pale blue cartoon (**C**). HA residues involved in the ligand binding are represented as lines.

The analysis of docked poses shows that all ligands adopt a similar binding mode, occupying the site with an extended conformation. Key interactions on the A/Parma/H1N1 RBS are H-bonds with Val120, Tyr80, Gln210, Lys206, Glu211, Asp174, and π-π stacking with Trp137. The binding geometries observed in the strain A/Roma/H1N1 are very similar and involve almost the identical residues described before: H-bonds with Val123, Ala125, Tyr83, Lys210, Glu215, and π-π stacking with Trp141. In the RBS of the A/Parma/H3N2, ligands find fewer H-bond contacts with Ser152, Thr151, Tyr114, and hydrophobic contacts with Trp169 and Leu210. In Figure 4, the binding geometries of compound **4** in the three binding sites are reported. Docked poses of the other ligands are available in the Supplementary Materials.

### 2.5. Pharmacokinetic Profile

To complete the profiling of identified hits, physicochemical and pharmacokinetic properties were calculated using QuikProp [49]. All compounds are compliant with the Lipinski’s Rule of five. The predicted oral bioavailability of our compounds is very high, with an oral absorption reaching at least 78%. The cell permeability, simulated by the CaCo model, is also good, while solubility should be improved, as well as the interference with the HERG potassium channel that raises concern for some ligands (Table 4).

## 3. Discussion

The outbreak of SARS-CoV-2 dramatically demonstrated the risks of a global viral spread and highlighted the crucial role of prevention. Influenza virus is a highly monitored pathogen in this context: it is diffused mainly in the avian population, and its spillover to humans could represent a severe threat [50,51]. Despite its global diffusion, only a few drugs are clinically available, with vaccination representing a fundamental tool to prevent seasonal Flu. The identification of an effective weapon is still an unmet goal. 

In this scenario, our aim was to identify new drug-like compounds with anti-Influenza activity toward both A/H1N1 and A/H3N2 strains, the subtypes commonly present in seasonal Flu. 

In a recent article, Hussein and coworkers identified a potent H1N1 inhibitor through a high throughput screening (HTS) of 19,200 compounds [52]; in 2014, Basu et al. published the identification of two strain-specific HA ligands through HTS of 106,000 compounds [53]. In the whole procedure applied, considering the previously published diverse library screening [41], we globally assayed 148 compounds and identified six broad-spectrum compounds with a final hit rate of 4%. 

In the search for effective anti-Influenza agents, we focused on HA. This large glycoprotein is the target of many studied compounds that mainly block the fusion machinery and interact with the stem portion of HA [54,55,56]. Because of its role in the internalization process, this region is less prone to mutations and, therefore, is a better target for broad-spectrum compounds. Fewer ligands were studied that bind HA occupying the RBS [57,58]. Computational studies accompanying the discovery of HA binders pose the main question on the exact ligand binding site positioning on the HA surface that has been largely investigated [59,60]. 

Our compounds proved to inhibit the hemagglutination caused by the interaction between viral hemagglutinin (HA) and surface glycoproteins on the target cells. This activity can be attributed to the interference with the recognition of the sialic acid in the RBS of HA. The docking studies confirmed the ability of these molecules to interact with residues at this shallow binding site. 

The same molecules were tested to verify their antiviral activity, showing a sub-micromolar potency and a promising selectivity index. In addition, the physicochemical properties indicate the excellent oral bioavailability of active compounds.

**Figure 5:**
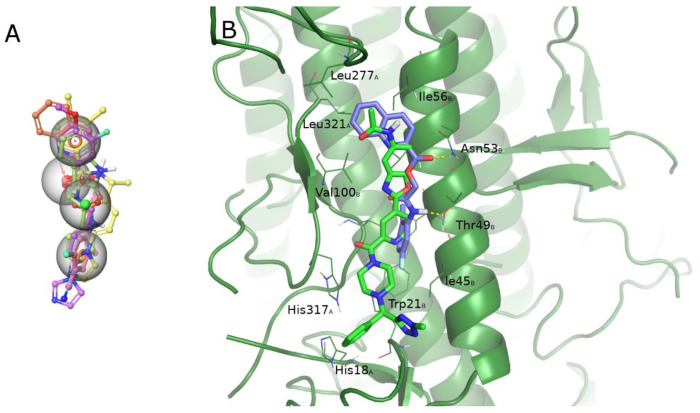
(**A**) The pharmacophore hypothesis obtained aligning the two X-ray ligands binding the stem region of HA, and characterized by two aromatic rings (orange rings), one H-bond acceptor (red sphere), and one hydrophobic (green sphere) feature. All six active ligands are aligned to the hypothesis matching all the features. (**B**) Docked pose of ligand **4** (violet C atoms) in the binding site on the stem region superimposed to the X-ray ligand (PDB ID 6CF7). HA (A/Parma/H1N1) is represented as a green cartoon. Residues involved in the ligand-binding are represented as lines; residue name is followed by a subscript indicating the HA chain.

Previously identified compounds (**1** and **4**) also resulted active in hemolysis assays toward A/H1N1 [41]. In the present work, this assessment was not carried out for newly discovered ligands, but we can envision for this latter a similar behavior of the hit analogues. Hemolysis inhibition stands for interference with hemagglutinin functions. It is well known that hemagglutinin is necessary for host membrane adhesion and virus internalization. This function is due to a conformational rearrangement of the protein triggered by low pH. As already mentioned, several known ligands can bind a conserved region (stem) of the HA close to the fusion peptide [33]. The structural analysis of these ligands highlights the similarity of these compounds with our inhibitors (Figure 5A). To better assess this possibility, a pharmacophore model was generated accounting for common structural features shared by X-ray ligands in complex with H1N1 HA (PDB ID: 6CF7 [55] and 6WCR [54]). We aligned our ligands to the obtained pharmacophore and retrieved a good alignment for all identified ligands (Figure 5A), suggesting the possibility for these compounds to interact with the stem region of HA. An induced-fit docking protocol was applied for compound **4** binding A/Parma/H1N1 HA to verify this hypothesis further. The binding site on the stalk region of HA was derived from recently resolved X-ray complexes of H1 HA bound to small molecules [54,55]. The ligands occupy the HA epitope recognized by universal antibodies on the HA stem in both cases. The same HA region is involved in the bovine lactoferrin binding in several binding poses we obtained through protein-protein docking in a previous study [37].

**Figure 6:**
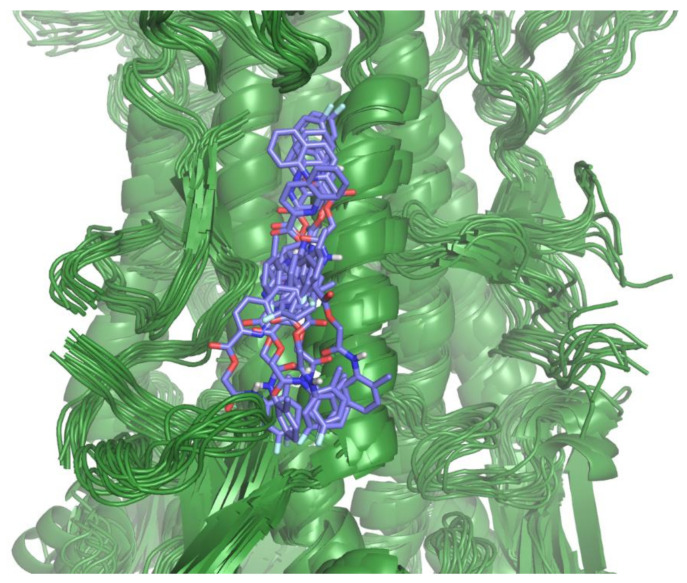
Alignment of the A/Parma/H1N1 HA and compound **4** complexes obtained after clustering the MD trajectory frames. Cluster representatives are reported.

Best binding geometry shows our ligands located in the same region of HA occupied by crystallographic ligands. In particular, they can reach a first hydrophobic patch surrounded by residues: Val100, Leu321 of chain A, Ile56, Leu277 of chain B where an aromatic function is placed, the H-bond contacts with Asn53 and Thr49 of chain B, and another hydrophobic patch characterized by His18, His317 of chain A and Trp21, Ile45 of chain B (Figure 5B) is only partially occupied. To further verify the binding hypothesis, a 50 ns MD simulation was carried out on the docked complex that confirmed the ligand maintaining its interaction on the HA stem, even exploring the cleft between HA1 and HA2 (Figure 6, more details about the MD simulation are available in Supplementary Materials). These results support the hypothesis of the possible role of our compounds as fusion peptide inhibitors, along with their ability to occupy the sialic acid-binding site. The hypothesized dual binding site is not new and was also demonstrated for cyclo-hexyl-taurine in a complex with H5 HA [48]. Further studies will be carried out to verify our hypothesis experimentally.

## 4. Materials and Methods

### 4.1. Compounds

Small molecules identified in the computational screen of ZINC database were purchased from Enamine Ltd. as dry powders. Compound purity, assessed by NMR and LC-MS, is higher than 92%. Chiral compounds are provided as racemic mixture. Compounds were dissolved in dimethyl-sulfoxide (DMSO, Sigma, Chemical Company, St. Louis, MO, USA) at 2 mmol/L and stored at −20 °C in amber glass vials.

### 4.2. Biological Assay

#### 4.2.1. Cells and Viral Strains

Madin-Darby canine kidney (MDCK, ATCC, CRL-2936) cells were grown at 37 °C in minimal essential medium (MEM, Invitrogen, Paisley, UK) containing 1.2 g/L NaHCO_3_, and supplemented with 10% inactivated fetal calf serum (FCS, Invitrogen, Paisley, UK), 2 mM glutamine, nonessential amino acids, penicillin (100 IU/mL), and streptomycin (100 μg/mL). 

The following Influenza A virus strains were used: A/RomaISS/02/08 H1N1 (Brisbane-like) oseltamivir-sensitive virus, A/Parma/24/09 H1N1 (Brisbane-like) oseltamivir-resistant virus, and A/Parma/05/06 H3N2 (Wisconsin-like). Viruses were propagated in MDCK cells in serum-free MEM supplemented with 4% Bovine Serum Albumin (BSA fraction V, Gibco; Paisley, UK), 1 µg/µL N-tosyl-L-phenylalanine chloromethyl ketone-treated trypsin (Sigma Chemical Co.; St. Louis, MO, USA). When extensive cytopathic effect (c.p.e.) was observed, infected cultures were frozen and thawed three times, centrifuged (3000 r, 10 min), and supernatants were stored at −80 °C. Titers of virus stocks were determined by hemagglutinin titration and/or plaque assay according to the standard procedures [61,62].

#### 4.2.2. Cytotoxicity Assay

This procedure was performed as reported elsewhere [41]. Briefly, two-fold serial dilutions of each compound and DMSO or H2O in culture medium were incubated at 37 °C with confluent MDCK cells grown in 96-well tissue culture microplates (Nalge Nunc Europe Ltd., Neerijse, Belgium). After 24 and 48 h, the following parameters were evaluated: cell morphology was examined by light microscopy, cell viability was determined by neutral red staining as already described by us [63], and cell proliferation was evaluated quantitatively by microscopic counts after dispersion into individual cells with trypsin. Compound dilutions that did not affect any of these parameters were considered as non-cytotoxic concentrations and utilized for antiviral assays.

#### 4.2.3. Hemagglutination Inhibition (HI) Assay

Viruses in PBS (Phosphate-buffered saline) were incubated for 1 h at 4 °C with serial dilutions of compounds in PBS. An equal volume of 0.5% turkey erythrocytes was then added and allowed to agglutinate. Titers were expressed as the reciprocal of the compound dilutions giving 50% hemagglutination of erythrocytes by four virus-agglutinating units. 

#### 4.2.4. Neutralization Assay

Neutralization was carried out by incubating serial two-fold compound dilutions, starting from 1 µM, in culture medium with equal volumes of viral suspension containing 10^6^ p.f.u. for 1 h at 4 °C. In negative controls, culture medium was used instead of compounds in the same volume. MDCK cells, grown in 96-well tissue culture microplates (Nalge Nunc Europe Ltd., Neerijse, Belgium), were infected with 100 μL/well (10 p.f.u./cell; in quadruplicate) of the virus-compound mixtures. After viral adsorption, cells were rinsed thoroughly and incubated at 37 °C for 48 h. The viral cytopathic effect (c.p.e.) was measured by neutral red staining as reported elsewhere by our laboratory [63].

### 4.3. Computational Studies

Computational studies were carried out using the Schrodinger Suite 2021-2 [49].

#### 4.3.1. Homology Modeling

Homology models for the three viral strains were generated using the Swiss Model web server (https://swissmodel.expasy.org/, accessed on 29 October 2020). Methods and quality parameters are reported in a previously published article [39]. 

#### 4.3.2. Protein Preparation

The three generated structures were aligned and submitted to the Protein Preparation routine in Maestro [49] to check the structure, optimize hydrogen atom position and H-bond network. A final restrained minimization was carried out.

#### 4.3.3. Binding Site Identification and Analysis

The homology models were aligned to the available X-ray complex of pandemic HA bound to 6′-SLN (PDB ID: 3UBN [64]) to locate the putative binding region on the receptor binding site of the studied HAs. This site was explored by performing a SiteMap [49] calculation. 

#### 4.3.4. Receptor Grid Generation

For the site mentioned in the previous paragraph, the receptor grid was generated following the procedure already mentioned [39].

#### 4.3.5. Ligand Preparation

The structures of the active ligands were generated with the Build tool in Maestro. All compound structures were prepared through Ligprep, which calculates tautomers and protomers at physiological pH (7.0 ± 0.4). The resulting structures were minimized to a derivative convergence of 0.001 kJÅ^−1^mol^−1^ using the PRCG minimization algorithm, the OPLS4 force field, and the generalized Born/surface area (GB/SA) water solvation model implemented in MacroModel [49].

#### 4.3.6. Docking Calculations

Minimized ligand structures were docked in the RBS on the HA surface using Glide [49,65] and the SP-peptide docking protocol as already described [39]. Briefly, the method allows for expanding the conformational exploration of ligands. To detect the most plausible binding geometry, all obtained poses were clustered applying the Kelley penalty to define the clustering level. Ligand geometries with the highest docking scores and in the most populated clusters were evaluated.

#### 4.3.7. Induced Fit

The induced fit calculation was carried out using the module available in Maestro that combines the Glide docking ability with the Prime homology modeling to adapt the protein to the ligand binding. The calculation was carried out on the A/Parma/H1N1, and the A/Roma/H1N1 HAs docking compound **4**. The grid in the stem region was centered on the X-ray ligand with PDB ID 6CF7, and default settings were applied for the remaining parameters.

#### 4.3.8. Molecular Dynamics

The best docked pose of compound **4** on the Parma/H1N1 HA stem was submitted to MD simulation using Desmond available in the Schrodinger Suite 2021-2 [49]. The system was prepared embedding the complex in a triclinic box of single point charge (SPC) water molecules. Fifteen Na ions were added to neutralize the system. A total of 148,519 atoms were simulated using the OPLS4 force field. The system was submitted to six relaxation steps as by default before the simulation. The production phase of the simulation lasted 50 ns, recording frames each 100 ps using a normal pressure temperature (NPT) ensemble with a Nose-Hoover thermostat at 300 K and Martyna-Tobias-Klein barostat at 1.01325 bar pressure. The electrostatic interactions were analyzed using the smooth Particle Mesh Ewald method. The trajectory frames were clustered based on the ligand coordinates. The representative complexes of the 12 obtained clusters were superimposed.

The Simulation Interaction Tool available in Maestro was used to check ligand protein contacts, RMSD and RMSF of protein and ligand (Supplementary Materials).

#### 4.3.9. Pharmacophore Alignment

A common pharmacophore hypothesis was built from the superimposed geometries of the two X-ray ligands [54,55] using Phase [49,66]. To align the six active ligands to the obtained hypothesis features, 50 conformers were generated for each structure.

## 5. Conclusions

To summarize, in the present work, we disclosed six new compounds endowed with broad-spectrum activity toward the Influenza A virus is in the sub-micromolar range. The compounds, besides, show a good pharmacokinetic profile, with an excellent predicted oral absorption, and are synthetically tractable. Therefore, further optimization will be easily carried out along with the testing on other critical viral strains, such as H5 and H7.

Computational studies verified the ability of identified molecules to bind the RBS of HAs and suggested the possible binding in the stem region of this glycoprotein. While this latter hypothesis needs experimental validation, antiviral activity showed by our ligands makes them a valuable tool to be exploited in the fight against the Influenza virus.

## Figures and Tables

**Table 1:** HI titers of compounds **1**–**5** on the three studied viral strains.

ID	Structure	HI Titer (μM)		
		A/Parma H1N1 ^a^	A/Roma H1N1 ^a^	A/Parma H3N2
**1**	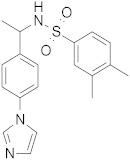	0.48	0.24	0.48
**2**	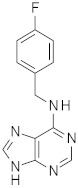	0.48	0.48	-
**3**	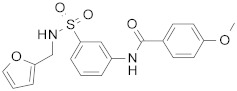	0.48	0.48	-
**4**	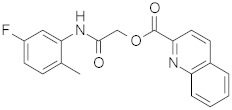	0.48	0.48	0.48
**5**	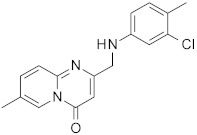	0.48	0.48	-

^a^ Data already published in the previous article [41].

**Table 2:** HI titers of the 23 active compounds toward at least one of the HA strains. Common substructure is highlighted in blue for ligand **1** analogues and in red for ligand **4** analogues.

ID	Structure	HI Titer (pM)			
		A/Roma H1N1	A/Parma H1N1	A/Parma H3N2	Ref Cmpd
**6**	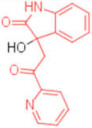	0.012	0.012	480	**4**
**7**	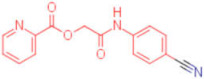	0.047	0.012	12,500	**4**
**8**	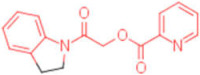	0.012	0.012	-	**4**
**9**	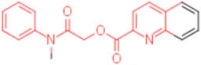	0.012	0.012	-	**4**
**10**	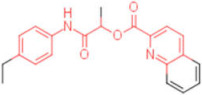	0.012	0.012	-	**4**
**11**	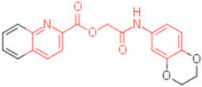	0.012	0.012	-	**4**
**12**	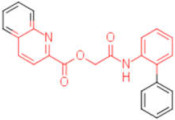	0.012	0.012	-	**4**
**13**	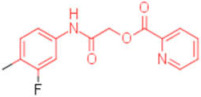	24	0.012	6	**4**
**14**	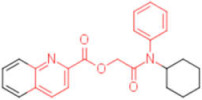	-	0.012	-	**4**
**15**	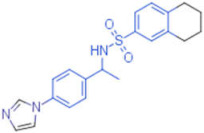	0.012	-	-	**1**
**16**	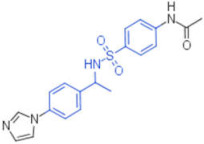	0.012	0.012	-	**1**
**17**	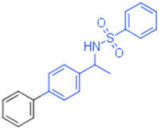	-	0.012	-	**1**
**18**	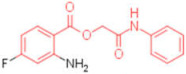	0.012	0.012	-	**4**
**19**	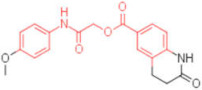	-	0.012	-	**4**
**20**	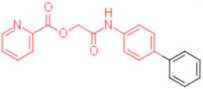	0.012	0.012	-	**4**
**21**	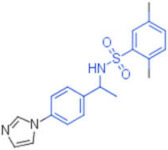	0.012	-	-	**1**
**22**	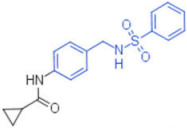	0.012	-	-	**1**
**23**	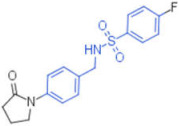	12	0.012	-	**1**
**24**	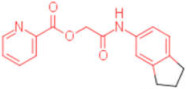	0.024	0.047	-	**4**
**25**	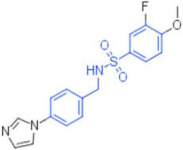	0.012	0.012	50,000	**1**
**26**	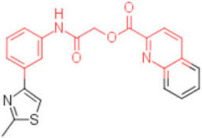	0.093	0.047	-	**4**
**27**	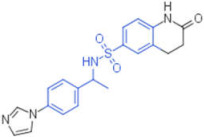	-	-	25,000	**1**
**28**	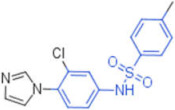	0.093	0.047	-	**1**

**Table 3:** In vitro antiviral activity of broad-spectrum compounds.

ID	CC_50_ *(μM)	A/Roma H1N1		A/Parma H1N1		A/Parma H3N2	
		EC_50_ (nM) ^∞^	SI ^^^	EC_50_ (nM) ^∞^	SI ^^^	EC_50_ (nM) ^∞^	SI ^^^
**1**	300	500 +/−0.01 *p* < 0.01 ^a^	6 × 10^2 a^	125 +/−0.01 *p* < 0.01 ^a^	2.4 × 10^3 a^	98 +/−0.01 *p* < 0.01	3 × 10^3^
**4**	250	62 +/−0.07 *p* < 0.01 ^a^	4 × 10^3 a^	125 +/−0.03 *p* < 0.01 ^a^	2 × 10^3 a^	125 +/−0.02 *p* < 0.01	2 × 10^3^
**6**	31.25	200 +/−0.001 *p* < 0.02	1.56 × 10^2^	900 +/−0.01 *p* < 0.01	0.34 × 10^2^	300 +/−0.001 *p* < 0.01	1.04 × 10^2^
**7**	31.25	800 +/−0.05 *p* < 0.01	0.39 × 10^2^	900 +/−0.003 *p* < 0.01	0.34 × 10^2^	700 +/−0.002 *p* < 0.01	0.44 × 10^2^
**13**	31.25	700 +/−0.04 *p* < 0.01	0.47 × 10^2^	700 +/−0.04 *p* < 0.01	0.44 × 10^2^	500 +/0.05 *p* < 0.01	0.62 × 10^2^
**25**	31.25	900 +/−0.02 *p* < 0.01	0.34 × 10^2^	700 +/−0.002 *p* < 0.01	0.44 × 10^2^	800 +/0.01 *p* < 0.01	0.39 × 10^2^

* CC50 cytotoxic concentration 50%, ^∞^ EC50 effective concentration 50%, ^^^ SI (selectivity index) = CC50/EC50. The mean values of 3 independent experiments with standard errors are shown. ^a^ Data already published in the previous article [41].

**Table 4:** Predicted pharmacokinetic properties of the most interesting compounds.

Cmpd	mol MW	QPlogPo/w ^a^	QPlogS ^b^	QPlogHERG ^c^	QPPCaco ^d^	#metab ^e^	QPlogKhsg ^f^	PercentHumanOralAbsorption	PSA ^g^
**1**	355.454	3.941	−5.442	−6.374	1074.843	3	0.405	100.000	60.952
**4**	338.337	3.947	−5.292	−6.437	1607.601	3	0.321	100.000	72.475
**6**	268.271	1.351	−2.292	−4.267	255.939	3	−0.307	77.956	97.449
**7**	288.278	2.779	−4.527	−6.168	670.015	3	−0.007	93.798	84.973
**13**	361.390	2.138	−2.551	−4.094	634.323	2	−0.215	89.620	72.606
**25**	281.270	1.431	−4.575	−6.339	150.454	2	−0.357	74.293	110.030

^a^ QPlogPo/w indicates the octanol/water partition coefficient; ^b^ QPlogS is the log of the water solubility expressed as molar concentration; ^c^ QPlogHERG indicates the ability to block the HERG potassium channel as IC_50_ (concern below −5); ^d^ QPPCaco stands for the Caco-2 cell permeability in nm/s (<25 poor, >500 great); ^e^ Number of likely metabolic reactions; ^f^ QPlogKhsg binding to human serum albumin (−1.5–1.5); ^g^ PSA Polar Surface Area.

## Data Availability

Structural data of representatives of clusters obtained from MD trajectory frames of the A/Parma/H1N1 HA and compound **4** complexes are available as Supplementary Materials.

## Supplementary Materials

The following are available online at https://www.mdpi.com/article/10.3390/ph15030301/s1, Figure S1: The scaffold of ligands 1 and 4 used for the substructure; Table S1: Structure and ID of tested analogs; Figure S2: Docked poses of compound **1** in the RBS of studied HAs; Figure S3: Docked poses of compound **6** in the RBS of studied HAs; Figure S4: Docked poses of compound **7** in the RBS of studied HAs; Figure S5: Docked poses of compound **13** in the RBS of studied HAs; Figure S6: Docked poses of compound **25** in the RBS of studied HAs; Figure S7: RMSD values for ligand 4 and protein; Figure S8: RMSF values calculated for HA residues; Figure S9: Most conserved contacts between ligand 4 and protein; Figure S10: Timeline representation of the ligand protein contacts; Figure S11: Type of interactions between ligand 4 and HA.
