# Supplementary material for: Broad-Spectrum Activity of Small Molecules Acting against Influenza a Virus: Biological and Computational Studies
Source: Pharmaceuticals (Basel). 2022 Feb 28;15(3):301. doi: 10.3390/ph15030301 (PMC8952214; doi:10.3390/ph15030301)
Supplement: Supplementary file 1 [file pharmaceuticals-15-00301-s001.zip › clustered_md_ParmaH1_4/pharmaceuticals-1553000-supplementary.pdf]

SUPPLEMENTARY MATERIAL

Broad-spectrum activity of Small Molecules Acting Against Influenza A Virus: biological and computational studies

Mariangela Agamennone,[a] Fabiana Superti[b*]

[a]Department of Pharmacy, University "G. d'Annunzio" of Chieti-Pescara, Via dei Vestini 31, 66100, Chieti, Italy

[b]National Centre for Innovative Technologies in Public Health, National Institute of Health, Viale Regina Elena 299, 00161, Rome, Italy

*Correspondence: fabiana.superti@iss.it; Tel.: +39 0649903149

Table of contents

Supplementary tables and figures:

**Figure S1**	The scaffold of ligands **1** and **4** used for the substructure	S2
**Table S1**	Structure of the 43 tested analogues	S3
**Figure S2**	Docked poses of compound **1** in the RBS of studied HAs	S4
**Figure S3**	Docked poses of compound **6** in the RBS of studied HAs	S5
**Figure S4**	Docked poses of compound **7** in the RBS of studied HAs	S6
**Figure S5**	Docked poses of compound **13** in the RBS of studied HAs	S7
**Figure S6**	Docked poses of compound **25** in the RBS of studied HAs	S8
**Figure S7**	RMSD values for ligand **4** and protein	S9
**Figure S8**	RMSF values calculated for HA residues	S9
**Figure S9**	Most conserved contacts between ligand **4** and protein	S10
**Figure S10**	Timeline representation of the ligand protein contacts	S10
**Figure S11**	Type of interactions between ligand **4** and HA	S10

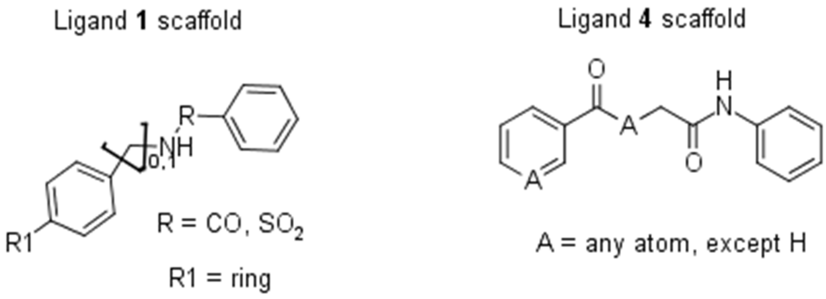

Figure S1. The scaffold of ligands **1** and **4** used for the substructure search in the Enamine database.

Table S1. Structure and ID of the 43 tested analogs.

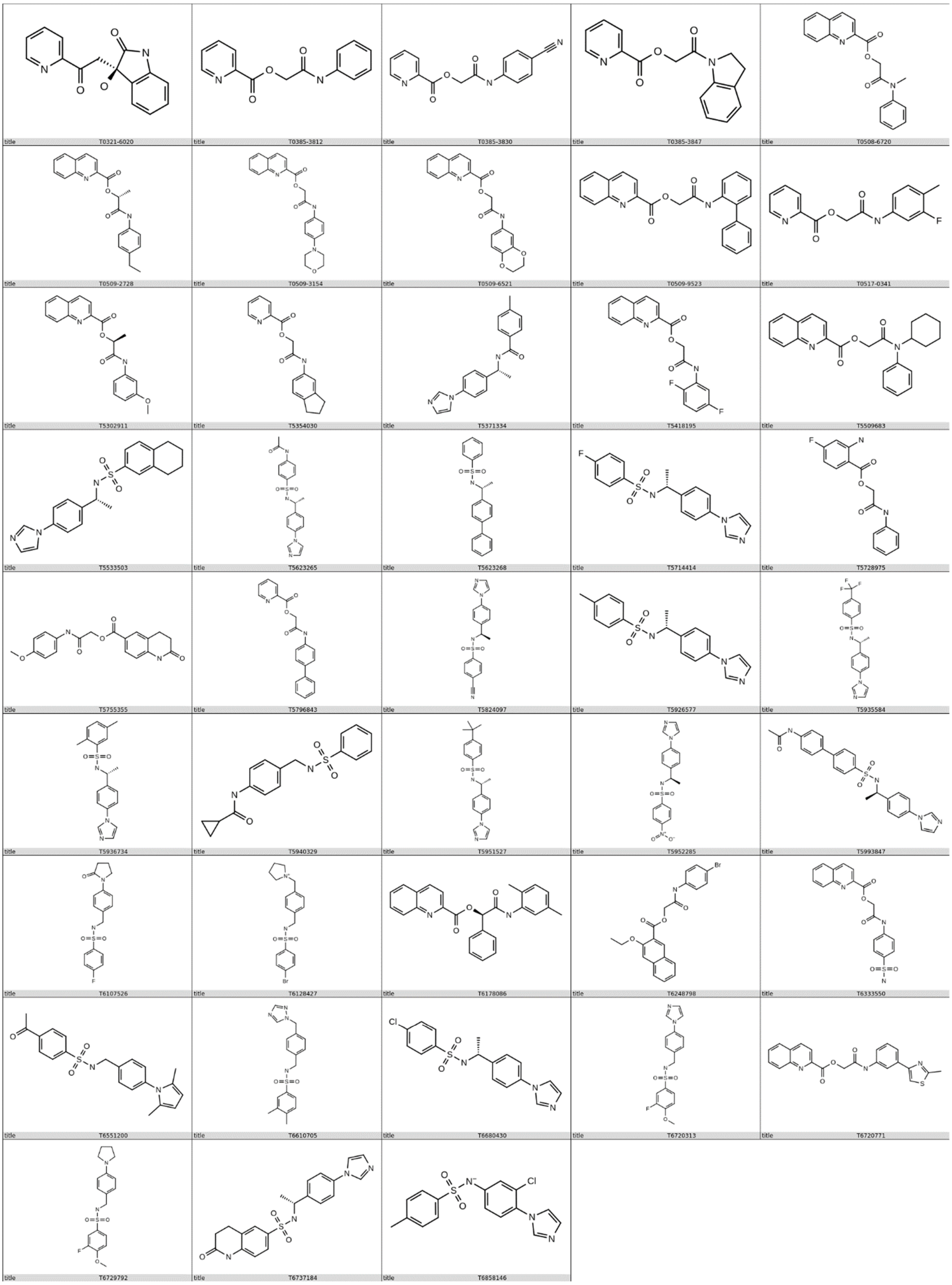

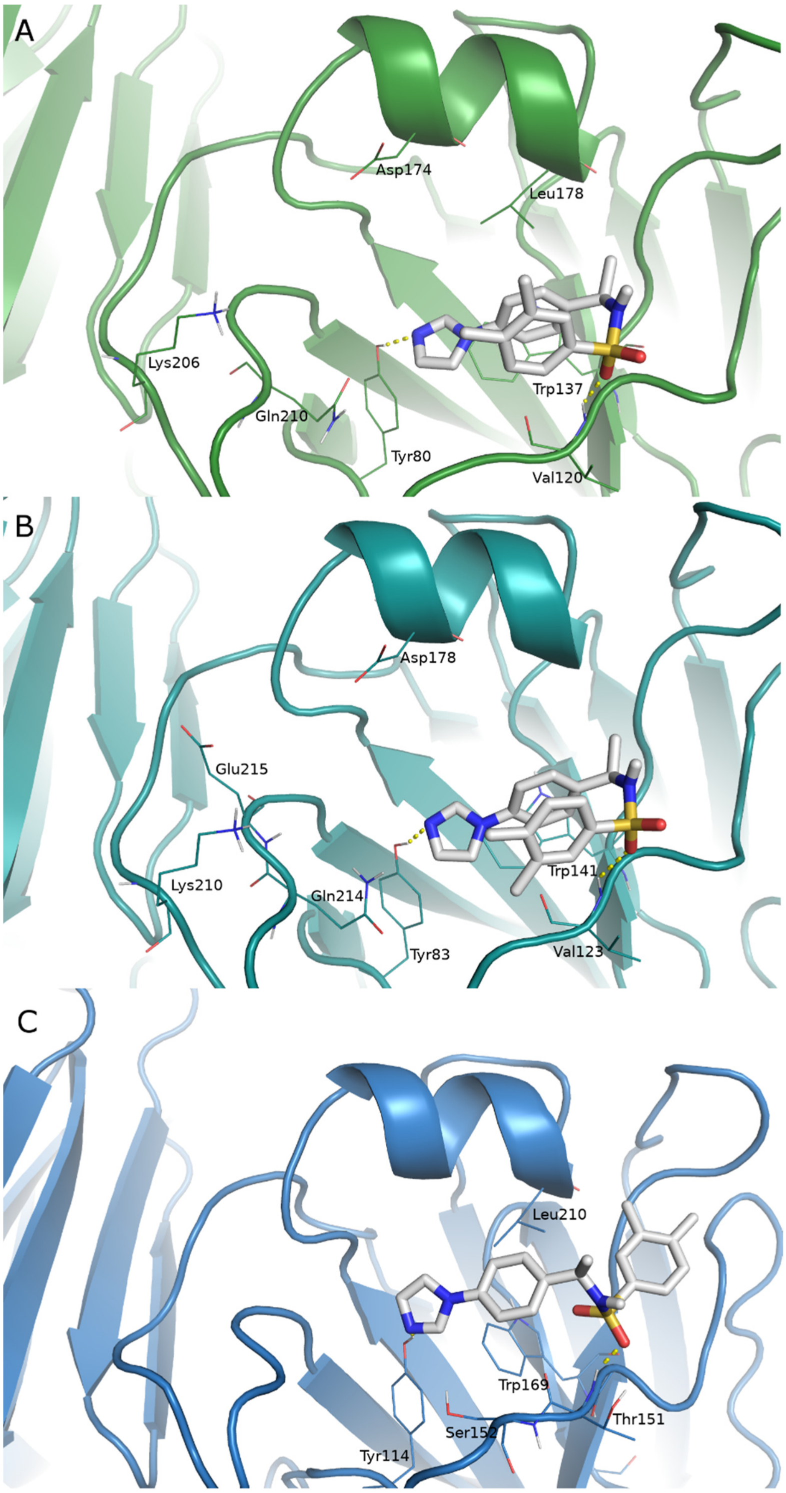

Figure S2. Docked pose of ligand **1** (stick, white C atoms) in the RBS of the HA of A/Parma/H1N1, green cartoon (A), A/Roma/H1N1, cyan cartoon (B), and A/Parma/H3N2, pale blue cartoon (C). HA residues involved in the ligand binding are represented as lines.

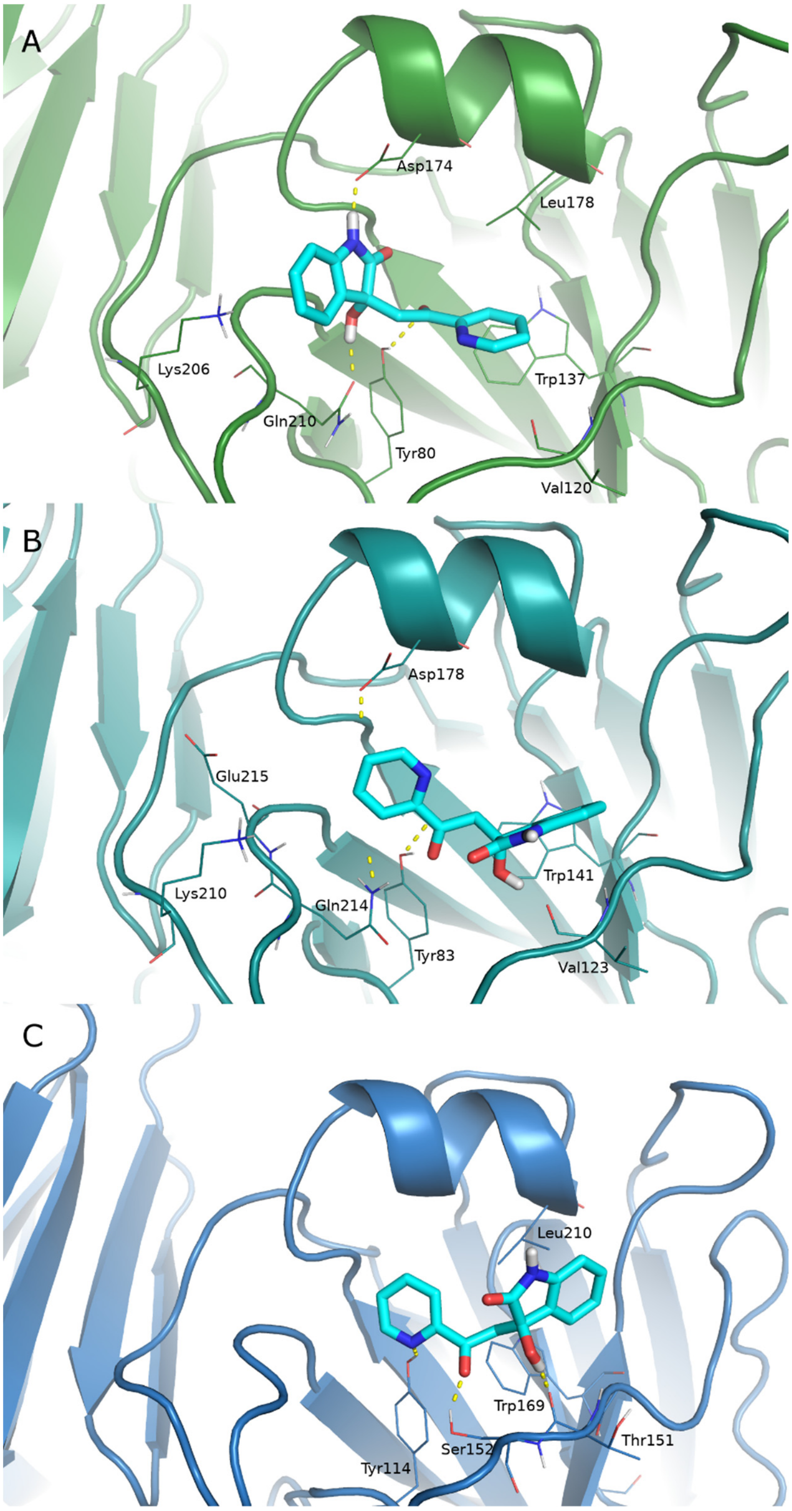

Figure S3. Docked pose of ligand **6** (stick, cyan C atoms) in the RBS of the HA of A/Parma/H1N1, green cartoon (A), A/Roma/H1N1, cyan cartoon (B), and A/Parma/H3N2, pale blue cartoon (C). HA residues involved in the ligand binding are represented as lines.

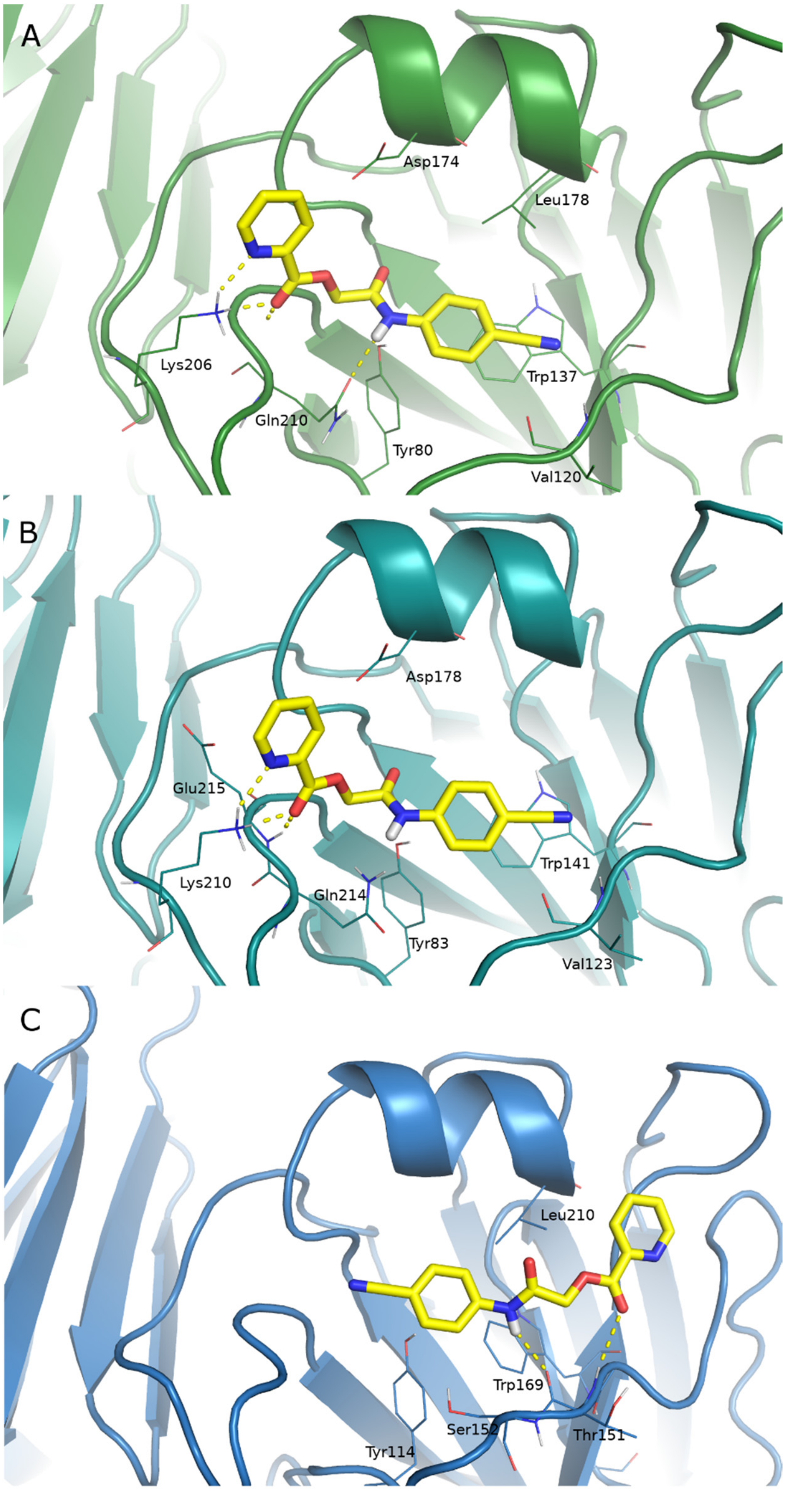

Figure S4. Docked pose of ligand **7** (stick, yellow C atoms) in the RBS of the HA of A/Parma/H1N1, green cartoon (A), A/Roma/H1N1, cyan cartoon (B), and A/Parma/H3N2, pale blue cartoon (C). HA residues involved in the ligand binding are represented as lines.

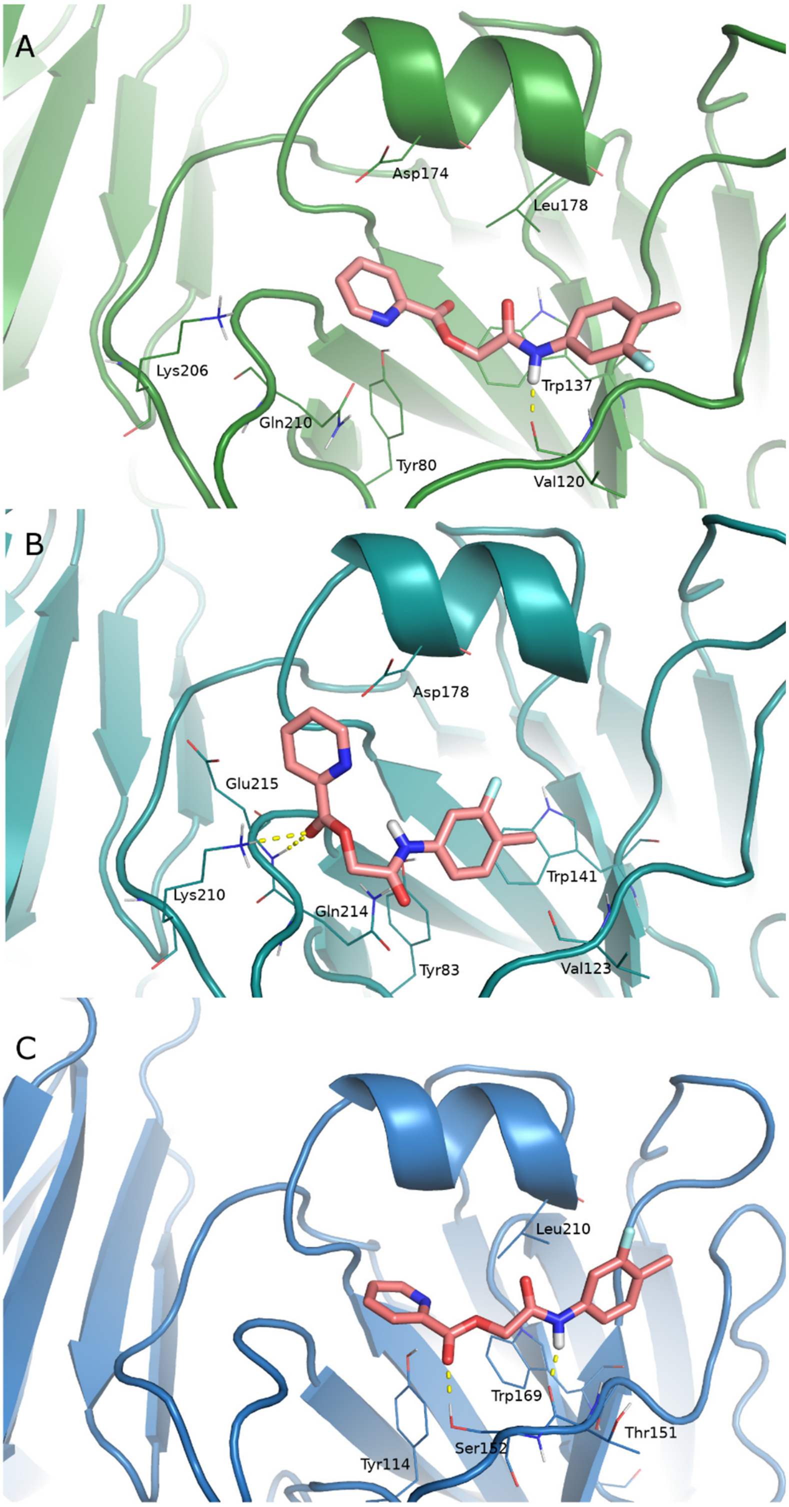

Figure S5. Docked pose of ligand **13** (stick, pink C atoms) in the RBS of the HA of A/Parma/H1N1, green cartoon (A), A/Roma/H1N1, cyan cartoon (B), and A/Parma/H3N2, pale blue cartoon (C). HA residues involved in the ligand binding are represented as lines.

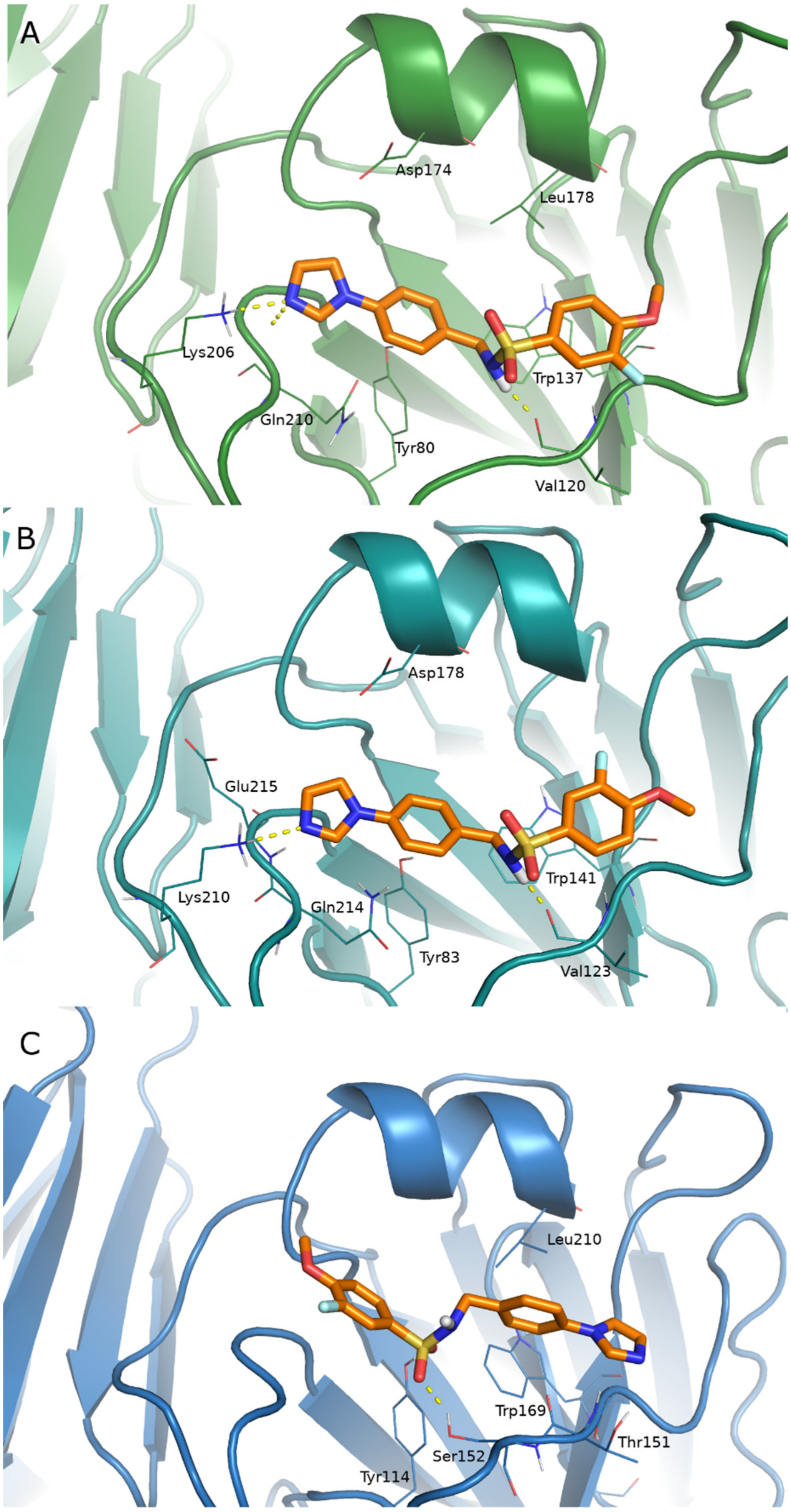

Figure S6. Docked pose of ligand **25** (stick, orange C atoms) in the RBS of the HA of A/Parma/H1N1, green cartoon (A), A/Roma/H1N1, cyan cartoon (B), and A/Parma/H3N2, pale blue cartoon (C). HA residues involved in the ligand binding are represented as lines.

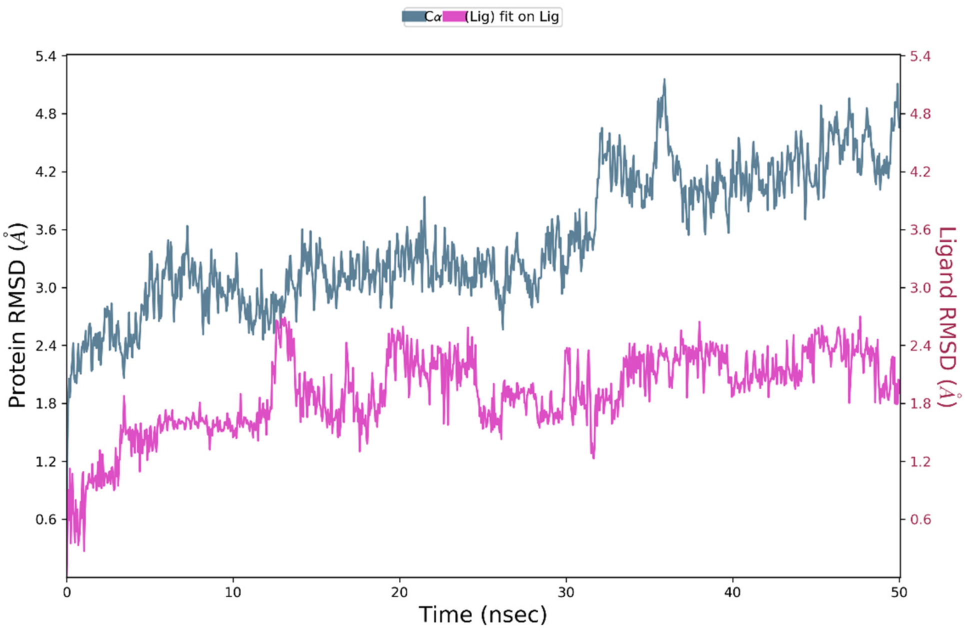

Figure S7. RMSD values for ligand **4** and protein C-alpha calculated along the simulation.

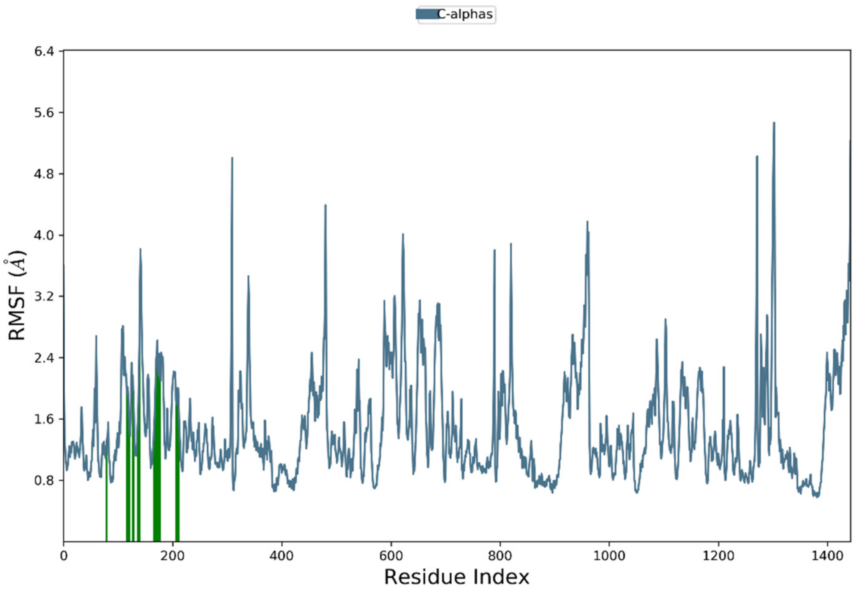

Figure S8. RMSF values calculated for HA residues.

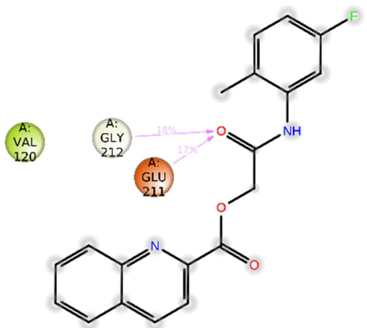

Figure S9. Most conserved contacts between ligand **4** and protein occurring along the trajectory.

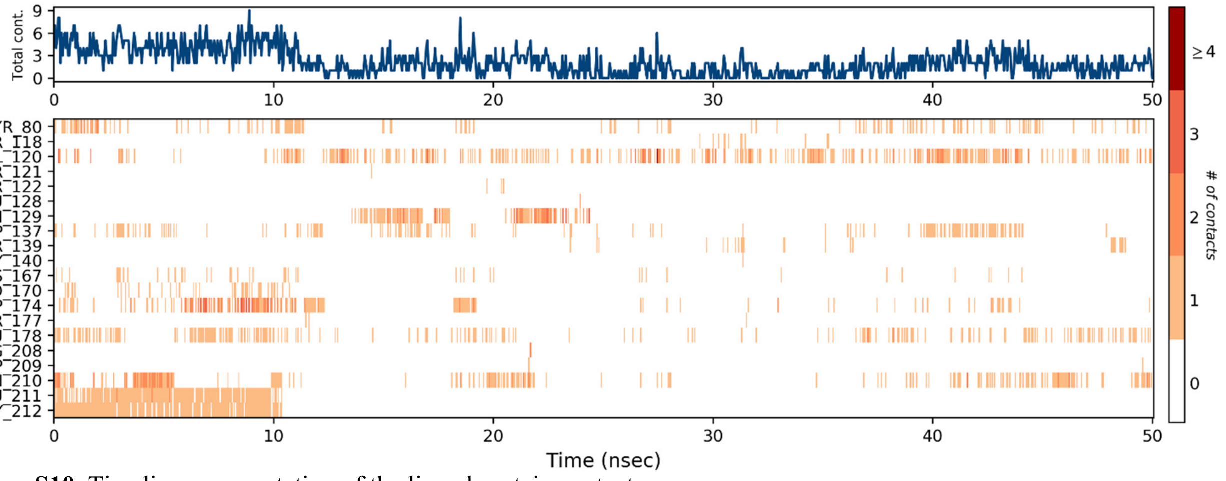

Figure S10. Timeline representation of the ligand protein contacts.

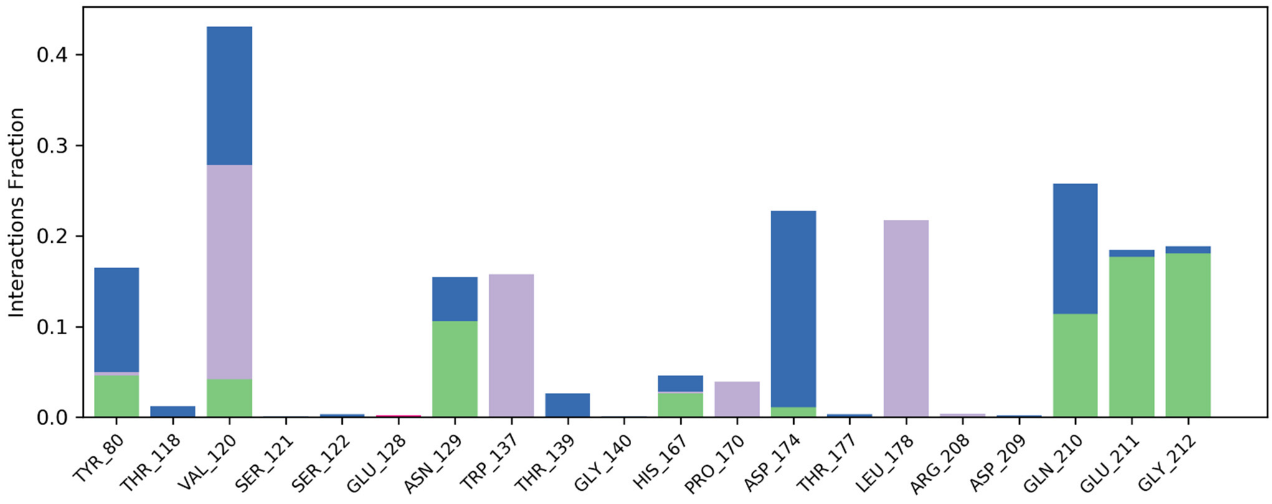

Figure S11. Type of interactions between ligand **4** and HA.
